# Reproductive Allocation in Three Macrophyte Species from Different Lakes with Variable Eutrophic Conditions

**DOI:** 10.1371/journal.pone.0165234

**Published:** 2016-11-02

**Authors:** Tao Wan, Qingxiang Han, Ling Xian, Yu Cao, Apudo A. Andrew, Xiaojie Pan, Wei Li, Fan Liu

**Affiliations:** 1 Key Laboratory of Southern Subtropical Plant Diversity, Fairy Lake Botanical Garden, Shenzhen & Chinese Academy of Science, Shenzhen 518004, P. R. China; 2 Sino-Africa Joint Research Center, CAS, Wuhan 430074, P. R. China; 3 Key Laboratory of Aquatic Botany and Watershed Ecology, The Chinese Academy of Sciences, Wuhan 430074, P. R. China; 4 Graduate School of Human and Environmental Studies, Kyoto University, Kyoto 606-8501, Japan; 5 Key Laboratory of Ecological Impacts of Hydraulic -Projects and Restoration of Aquatic Ecosystem of Ministry of Water Resources. Institute of Hydroecology, MWR&CAS, Wuhan 430074, China; University of Hyogo, JAPAN

## Abstract

Reproductive allocation is a key process in the plant life cycle and aquatic plants exhibit great diversity in their reproductive systems. In the present study, we conduct a field investigation of three aquatic macrophytes: *Stuckenia pectinata*, *Myriophyllum spicatum*, and *Potamogeton perfoliatus*. Our results showed that widespread species, including *S*. *pectinata* and *M*. *spicatum* had greater plasticity in their allocation patterns in the form of increased sexual and asexual reproduction, and greater potential to set seeds and increase fitness in more eutrophic environments. *P*. *perfoliatus* also exhibited a capacity to adopt varied sexual reproductive strategies such as setting more offspring for the future, although only in clear conditions with low nutrient levels. Our results establish strategies and mechanisms of some species for tolerating and surviving in varied eutrophic lake conditions.

## Introduction

Aquatic plants are a fascinating group for both naturalists and plant biologists, not only because of their special evolutionary significance but also for their ecological functions [1, 2]. At an ecological scale, they are critical components of shallow aquatic ecosystems, ensuring the health of these environments while forming the foundation of lake biodiversity [3, 4]. Investigations on aquatic plants are more complex when compared those on their terrestrial counterparts. The selective forces acting on terrestrial species vary a great deal from those in aquatic environments. Aquatic plants have to contend with relatively more complex physico-chemical and biological conditions [5, 6]. Since various groups of aquatic plants (systematic grouping) evolved over different time scales [5–7], the selective forces that drive their survival in aquatic conditions with different strategies potentially vary as well.

Aquatic plants have to adopt both physiological and morphological mechanisms in order to adapt and survive in aquatic environments. Plants could exhibit varying physiological mechanisms or responses, for example, varied tolerance levels to nutrient toxicity. Eutrophication, therefore, could alter the N and P quantities, and light accessible to submersed macrophytes. Consequently, in an aquatic environment with a large Secchi depth gradient, light could be presumed to the greatest driver of the change in community or plant characteristics. Competitive responses to changes in light conditions are often rapid growth or plastic responses towards a taller habit [8–11]. This indicates that variations in conditions, for example light, could benefit some species while impairing the growth of others [12]. Physiological stress in eutrophic environments could also arise due to NH_4_^+^-N toxicity, another key driver of decline in macrophytes in high nutrient conditions [13–15]. NH_4_^+^-N toxicity could inhibit photosynthesis and growth [16,17], while influencing species distribution [9, 18]. Many studies have demonstrated the above [2, 19].

In recent times, besides reproduction mechanisms, allocation between sexual and asexual reproduction has provided insights on modes of plant adaptation to the aquatic environments. Aquatic plants adopt varied reproductive allocation strategies when under different stress factors associated with the aquatic environment. Asexual reproduction in aquatic plants is often associated with environments with fewer stressors or stable conditions, while sexual reproduction in often linked to and deemed beneficial in highly stressful or heterogeneous conditions [20, 21]. Generally, aquatic plants demonstrate a greater capacity for asexual reproduction compared to terrestrial species.

Most aquatic plants possess either non-specialized vegetative propagules in the form of either shoot fragments or specialized organs such as corms, rhizomes, tubers, stolons, and turions. In the absence of stressful factors, asexual reproduction may facilitate rapid propagation and population establishment and expansion [19]. Under highly variable environmental conditions, however, especially under dynamic selective regimes, vegetative reproduction may be inadequate in establishing long-term adaptation. Even a short-lived extreme event could threaten an entire plant population owing to the unsustainability of vegetative propagules in aquatic environment conditions [22]. In such cases, sexual reproduction is favored since it may produce genetic variation and recombination, which could facilitate survivorship of a plant population in the future [19, 23].

While aquatic plants may demonstrate varied reproductive allocation patterns under different eutrophic conditions, such patterns may not be consistent across plant species. In natural aquatic ecosystems, varied species may demonstrate different reproductive allocation patterns and capacities in the face of environmental stresses. Some species could also survive more stressful and more variable conditions, compared to others. Until recently, most investigations have focused on physiological mechanisms of tolerating physical and chemical stress factors to demonstrate and explain survival in numerous stressful aquatic environments [12, 13, 24]. It is necessary to explore other aspects, such as reproduction, through which aquatic plants could survive stressful environments, to reveal more mechanisms and strategies through which some aquatic plants survive stressful conditions [2].

In the present study, we carry out a field investigation on three aquatic macrophytes, *Stuckenia pectinate*, *Myriophyllum spicatum*, and *Potamogeton perfoliatus*, in lakes with different nutrient levels, with the aim of reporting evidence of survival facilitation through variable reproductive allocation patterns.

## Materials and Methods

### Study species

*Stuckenia pectinata*, *Myriophyllum spicatum*, and *Potamogeton perfoliatus* are submergent aquatic species found in aquatic fresh or brackish water lakes, ponds, rivers, channels, and marshes. The species may produce sexually via seeds, or asexually via shoot fragments and rhizomes [25–27]. *S*. *pectinata* and *M*. *spicatum* are widespread species found inhabiting lakes with varied nutrient statuses, including heavily eutrophicated lakes, while *P*. *perfoliatus* may only be found in lakes with clear waters.

### Materials and data collection

**Fig 1:**
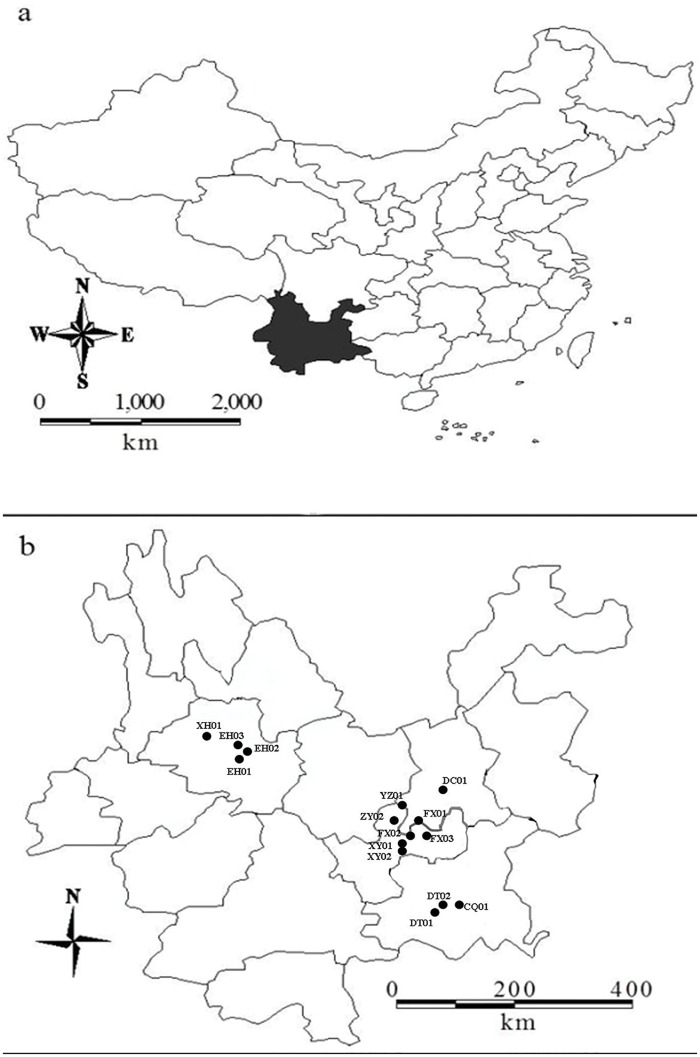
Sampling sites of the 8 lakes.

*S*. *pectinata*, *M*. *spicatum* and *P*. *perfoliatus* individuals were collected at 1.5 m depths in 14 sampling sites in 8 lakes in Yunnan province, China, from May to July 2015 (Fig 1, Table 1). All sampling sites and our sampling activities for which specific permission was not required and our field studies did not involve endangered or protected species. Physico-chemical data, including pH, TDS, and SAL, were measured at each sampling site (Table 1). TN, TP, chlorophyll-*a* (chl-*a*), and transparency of the lake waters were measured to determine the nutrient levels.

**Table 1:** Sampling sites, physicochemical parameters of the 8 lakes and presence and absence the three macrophytes species: *Stuckenia pectinata*, *Myriophyllum spicatum*, and *Potamogeton perfoliatus*.

Sampling sites	Lakes	Location	*Stuckenia pectinata*	*Myriophyllum spicatum*	*Potamogeton perfoliatus*	PH	TDS(mg/L)	SAL(ppt)
FX01	Fuxian Lake	N 24°37′30″, E 102°51′9″	+	+	+	8.41	230.1	0.17
FX02	Fuxian Lake	N 24°24′52″, E 102°50′17″		+		8.74	227.5	0.17
FX03	Fuxian Lake	N 24°26′26″, E 102°54′7″	+		+	8.52	228.2	0.17
EH01	Erhai Lake	N 25°54′1″, E 100°12′8″			+	8.90	217.7	0.16
EH02	Erhai Lake	N 25°55′56″, E 100°8′25″	+	+		9.14	218.4	0.16
YZ01	Yangzong Lake	N 24°51′35″, E 102°58′56″			+	8.68	303.5	0.22
YZ02	Yangzong Lake	N 24°52′7″, E 102°58′50″			+	8.98	297.7	0.22
XH01	Xihu Lake	N 26°11′3″, E 100°3′7″	+			8.75	317.8	0.24
DT01	Dadun Lake	N 23°26′4″, E 103°19′27″	+	+		8.40	409.6	0.31
DT02	Dadun Lake	N 23°24′60″, E 103°19′14″	+	+		8.30	409.5	0.30
CQ01	Changqiao Lake	N 25°25′47″, E 103°23′29″		+		8.92	302.9	0.22
DC01	Dianchi Lake	N 24°58′43″, E 102°37′59″	+	+		9.67	290.5	0.21
XY01	Xingyun Lake	N 24°22′42″, E 102°48′27″	+	+		9.32	481.9	0.36
XY02	Xingyun Lake	N 24°48′27″, E 102°46′18″	+	+		9.35	481.0	0.36

### Eutrophic evaluation

To evaluate the stress levels in the different environments, we apply the TLI method with some modifications. TN, TP, chl *a* and transparency (Secchi depths: SD) represent parameter j for evaluating lake nutrient and eutrophication levels [28]. The greater the TLI value, the more eutrophicated the lake is. Consequently, the 14 sampling sites provide 14 variable nutrient data for use in the following index:

TLI(∑)=∑wj•TLI(j)

TLI(∑): comprehensive trophic level index
*Wj*: the weighting of the comprehensive trophic level index of the parameter *j*.
TLI (*j*): the comprehensive trophic level index of the parameter *j*.

The variable parameter j calculation formulae are as follows [28]:

TLI(chl)=10(2.5+1.086 lnchl)

TLI(TP)=10(9.436+1.624 lnTP)

TLI(TN)=10(5.453+1.694 lnTN)

TLI(SD)=10(5.188−1.94 lnSD)

### Materials treatment

Following collection of plant individuals from the lakes, flower number, inflorescence number, and seed set number were determined. Afterwards, the individuals’ sexual (flowers and seeds) and asexual (leaves, shoots, and shoot fragments) structures were sorted and dried for two days in a desiccator at 50°C and weighed to the nearest 0.001 g.

### Data analysis

After calculating the TLI values, regressions between absolute sexual biomass, asexual biomass, ratio of total biomass allocation, and flower production were employed in comparisons between the reproductive components and degree of eutrophication based on the water TLI values. Afterwards, regressions between absolute biomass, asexual biomass, ratios of biomass allocation, and flower production were applied in comparisons between the reproductive components and environmental variables (TN, TP, Chla and Secchi). All data was analyzed in SPSS Statistics 19 (IBM) and figures plotted and fitted using Origin 8.0 (OriginLab, Northampton, MA).

## Results

The eutrophic levels of the sampling sites in the 8 lakes evaluated by the TLI method revealed that Fuxian (FX01, FX02, FX03), Erhai (EH01, EH02, EH03), and Yanzhong (YZ01, YZ02) lakes were clear water lakes. Dianchi (DC01) and Xingyun (XY01, XY02) lakes were heavily eutrophic lakes, while Xihu (XH01), Datun (DT01, DT02), and Changqiao (CQ01) were mesotrophic, representing medium state levels (Table 2).

**Table 2:** Nutrient and eutrophication levels in the sampling sites in 8 lakes based on TLI evaluation methods.

Sampling sites	TLI(TN)	TLI(TP)	TLI(chla)	TLI(SD)	w(TN)	w(TP)	w(chla)	w(SD)	total
FX02	41.58	1.16	36.34	43.31	0.14	0.00	0.12	0.15	16.79
FX03	36.14	14.32	36.69	47.64	0.12	0.05	0.13	0.16	17.47
EH02	37.18	33.31	52.00	47.64	0.13	0.11	0.18	0.16	25.44
FX01	20.64	40.08	52.05	66.67	0.07	0.14	0.18	0.23	31.30
EH01	49.00	43.41	51.05	51.18	0.17	0.15	0.17	0.17	32.40
YZ02	54.50	38.12	57.90	43.31	0.19	0.13	0.20	0.15	32.89
EH03	49.97	48.52	59.18	42.06	0.17	0.17	0.20	0.14	34.49
YZ01	53.58	44.85	59.38	42.06	0.18	0.15	0.20	0.14	34.67
XH01	63.85	56.24	75.52	65.02	0.22	0.19	0.26	0.22	58.50
DT02	75.87	60.94	73.01	73.29	0.26	0.21	0.25	0.25	68.72
DT01	72.03	56.91	75.68	82.40	0.25	0.19	0.26	0.28	71.35
CQ01	72.10	55.54	76.57	89.32	0.25	0.19	0.26	0.30	75.37
DC01	82.66	82.75	93.03	87.98	0.28	0.28	0.32	0.30	102.46
XY01	92.44	92.71	97.66	87.98	0.31	0.32	0.33	0.30	117.26
XY02	106.63	106.87	110.83	82.40	0.36	0.36	0.38	0.28	142.62

**Fig 2:**
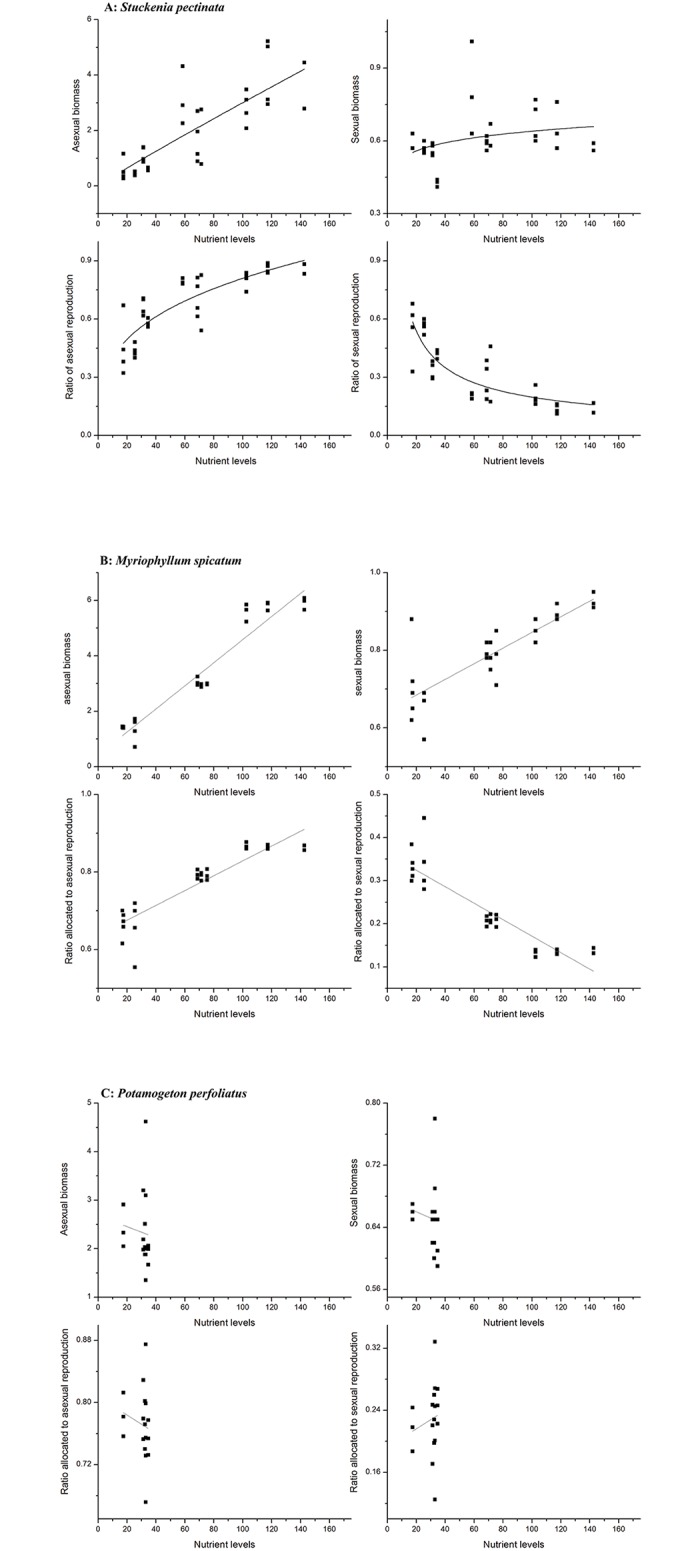
Regression between asexual biomass and nutrient levels; sexual biomass and nutrient levels and sexual/vegetative ratios and entrophic levels of three species A: *Stuckenia pectinata*, B: *Myriophyllum spicatum*, C: *Potamogeton perfoliatus*.

**Fig 3:**
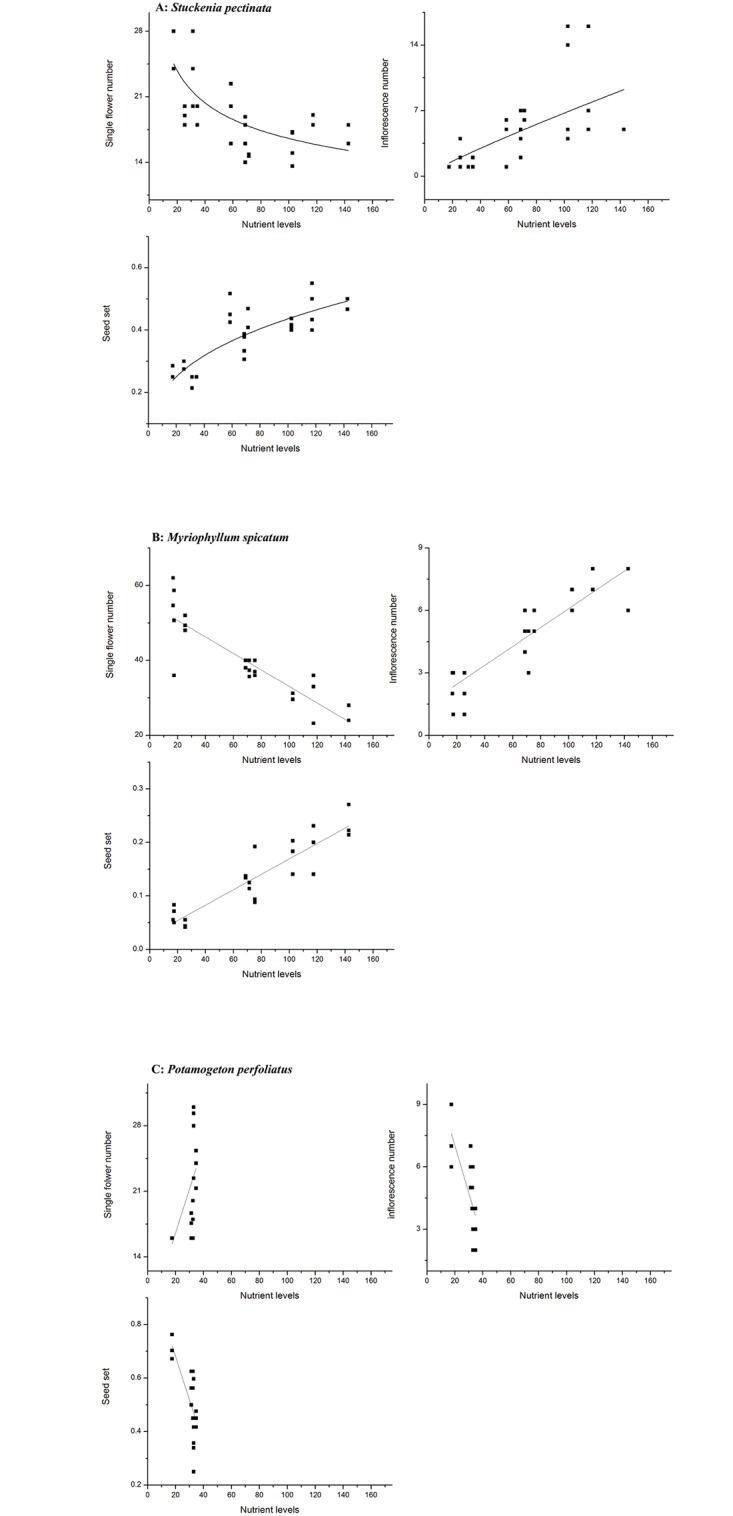
Regression between single flower number and nutrient levels; inflorescence number and nutrient levels and seed set and entrophic levels levels of three species A: *Stuckenia pectinata*, B: *Myriophyllum spicatum*, C: *Potamogeton perfoliatus*.

**Fig 4:**
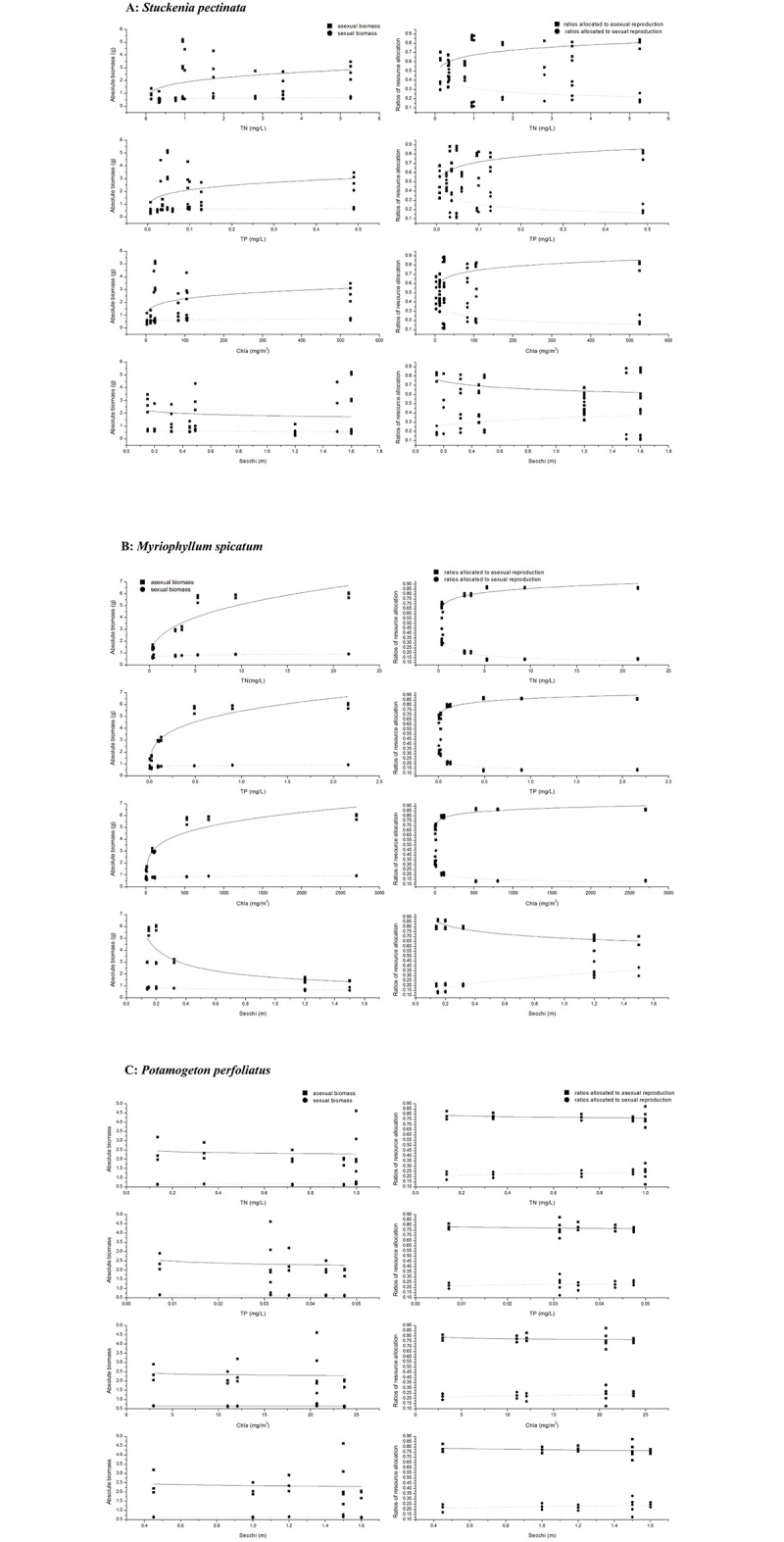
Regression between asexual biomass and nutrient levels; sexual biomass and nutrient levels and sexual/vegetative ratios and environmental variables (TN, TP, Chla and Secchi) of the three species A: *Stuckenia pectinata*, B: *Myriophyllum spicatum*, C: *Potamogeton perfoliatus*.

**Fig 5:**
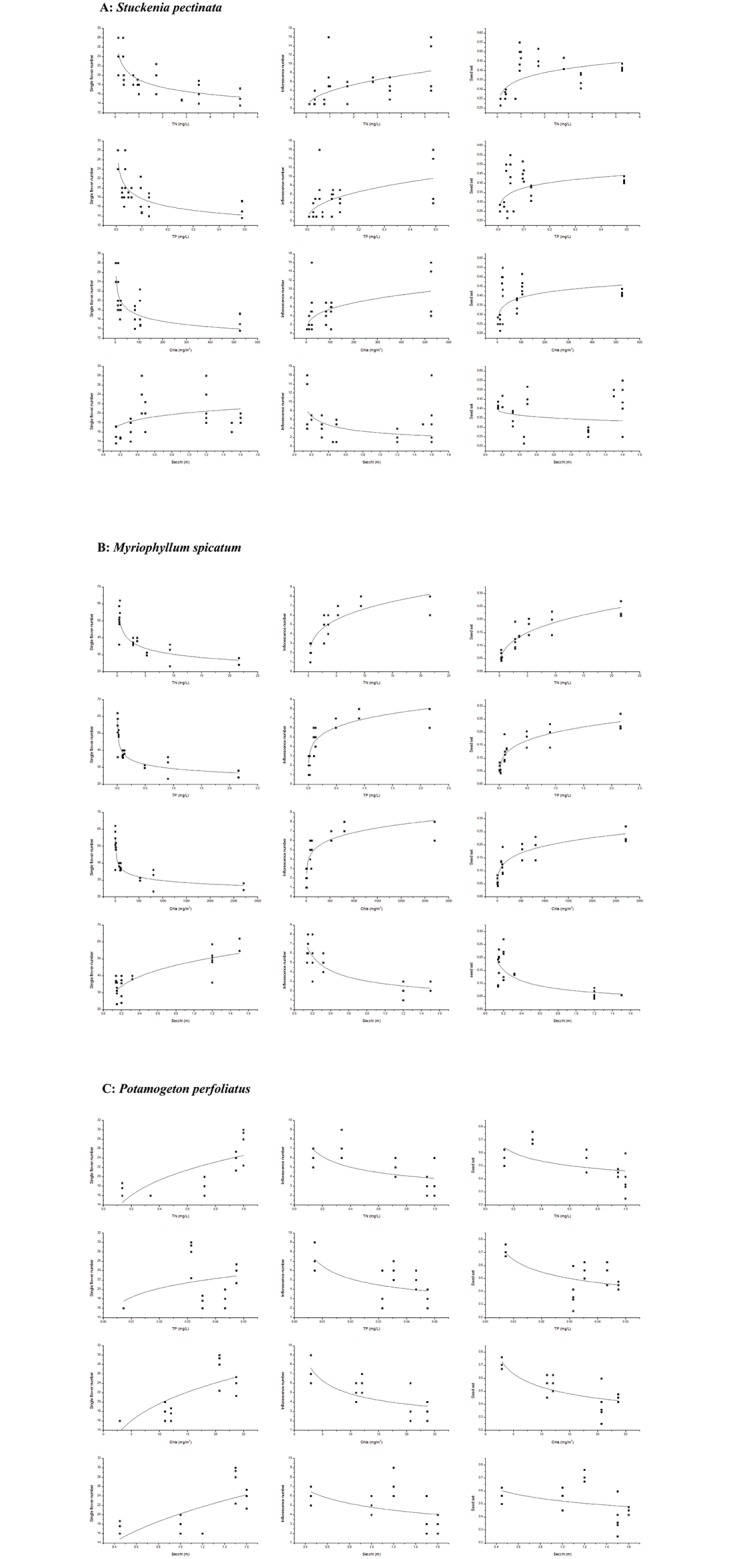
Regression between single flower number and nutrient levels; inflorescence number and nutrient levels and seed set and entrophic levels levels of three species A: *Stuckenia pectinata*, B: *Myriophyllum spicatum*, C: *Potamogeton perfoliatus*.

Absolute asexual biomass in *S*. *pectinata* increased with increase in nutrient levels, while sexual biomass did not increase. The ratio of asexual reproduction also increased with increase in eutrophic levels while ratio of asexual reproduction decreased (Fig 2a, Table 3). Flower and seed production were also examined, with flower number per inflorescence decreasing and inflorescence number and seed set increasing with increase in eutrophic levels (Fig 3a, Table 4). The relationship between reproductive components and environmental variables indicated that the asexual biomass, sexual biomass, ratios of sexual/asexual reproduction, flower and seed production were all correlated with the three environmental variables (TN, TP, Chla). Asexual biomass and ratio of asexual reproduction increased while the sexual biomass and ratio of sexual reproduction decreased with increase in TN, TP and Chla. Nevertheless, there were minimal changes in absolute biomass and ratio of biomass allocation with increase in Secchi depth (Fig 4a, Table 5). Flower number per inflorescence decreased and inflorescence number and seed set increased with increase in TN, TP and Chla. Additionally, Secchi depth had minimal effect on flower number per inflorescence, inflorescence number and seed set in *S*. *pectinata* (Fig 5a, Table 6).

**Table 3:** Regression analyses between asexual biomass and eutrophic levels; sexual biomass and eutrophic levels and sexual/vegetative ratios and eutrophic levels of three species.

	r^2^	*F*	*P*
*Stuckenia pectinata*			
Sexual biomass	0.075	573.39	< 0.001
Asexual biomass	0.645	113.03	< 0.001
Sexual ratio	0.704	256.30	< 0.001
Asexual ratio	0.690	925.95	< 0.001
*Myriophyllum spicatum*			
Sexual biomass	0.918	292.95	< 0.001
Asexual biomass	0.690	58.75	< 0.001
Sexual/Asexual ratio	0.814	115.06	< 0.001
*Potamogeton perfoliatus*			
Sexual biomass	0.058	0.13	0.728
Asexual biomass	0.052	0.21	0.652
Sexual/Asexual ratio	0.039	0.40	0.538

**Table 4:** Regression analyses between single flower number and eutrophic levels; inflorescence number and eutrophic levels, and seed set and eutrophic levels of three species.

	r^2^	*F*	*P*
*Stuckenia pectinata*			
Single flower number	0.501	783.55	< 0.001
Inflorescence number	0.405	44.04	< 0.001
Seed set	0.696	778.58	< 0.001
*Myriophyllum spicatum*			
Single flower number	0.790	98.72	< 0.001
Inflorescence number	0.819	118.53	< 0.001
Seed set	0.835	132.08	< 0.001
*Potamogeton perfoliatus*			
Single flower number	0.263	6.72	0.020
Inflorescence number	0.429	13.02	0.003
Seed set	0.473	15.35	0.001

**Table 5:** Regression analyses between asexual/sexual biomass and environmental factors (TN, TP, Chla and Secchi); ratios allocated to asexual/sexual biomass and environmental factors (TN, TP, Chla and Secchi) of three species.

	TN		TP		Chla		Secchi	
	*R*^*2*^	*P*	*R*^*2*^	*P*	*R*^*2*^	*P*	*R*^*2*^	*P*
*Stuckenia pectinata*								
Asexual biomass	0.147	< 0.001	0.117	< 0.001	0.146	< 0.001	-0.019	< 0.001
Sexual biomass	0.101	< 0.001	0.072	< 0.001	0.148	< 0.001	0.106	< 0.001
Ratio allocated to asexual biomass	0.235	< 0.001	0.292	< 0.001	0.293	< 0.001	0.058	< 0.001
Ratio allocated to sexual biomass	0.223	< 0.001	0.3611	< 0.001	0.365	< 0.001	0.047	< 0.001
*Myriophyllum spicatum*								
Asexual biomass	0.844	< 0.001	0.895	< 0.001	0.892	< 0.001	-0.628	< 0.001
Sexual biomass	-0.715	< 0.001	-0.608	< 0.001	-0.638	< 0.001	0.483	< 0.001
Ratio allocated to asexual biomass	0.815	< 0.001	0.776	< 0.001	0.800	< 0.001	-0.777	< 0.001
Ratio allocated to sexual biomass	-0.848	< 0.001	-0.766	< 0.001	-0.800	< 0.001	0.787	< 0.001
*Potamogeton perfoliatus*								
Asexual biomass	-0.059	< 0.001	-0.008	< 0.001	-0.063	< 0.001	-0.062	< 0.001
Sexual biomass	-0.055	< 0.001	-0.048	< 0.001	-0.066	< 0.001	-0.040	< 0.001
Ratio allocated to asexual biomass	-0.012	< 0.001	-0.045	< 0.001	-0.030	< 0.001	-0.025	< 0.001
Ratio allocated to sexual biomass	-0.012	< 0.001	-0.045	< 0.001	-0.029	< 0.001	-0.025	< 0.001

**Table 6:** Regression analyses between single flower number and environmental factors (TN, TP, Chla and Secchi); inflorescence number and environmental factors (TN, TP, Chla and Secchi) and seed set and environmental factors (TN, TP, Chla and Secchi) of three species.

	TN		TP		Chla		Secchi	
	*R*^*2*^	*P*	*R*^*2*^	*P*	*R*^*2*^	*P*	*R*^*2*^	*P*
*Myriophyllum spicatum*								
Single flower number	0.7437	< 0.001	0.7951	< 0.001	0.7839	< 0.001	0.6785	< 0.001
Inflorescence number	0.7756	< 0.001	0.7491	< 0.001	0.7683	< 0.001	0.6912	< 0.001
Seed set	0.8299	< 0.001	0.8027	< 0.001	0.8112	< 0.001	0.5436	< 0.001
*Stuckenia pectinata*								
Single flower number	0.5032	< 0.001	0.5128	< 0.001	0.5550	< 0.001	0.0951	< 0.001
Inflorescence number	0.3129	< 0.001	0.2991	< 0.001	0.3140	< 0.001	0.1283	< 0.001
Seed set	0.3434	< 0.001	0.1801	< 0.001	0.2711	< 0.001	0.0048	< 0.001
*Potamogeton perfoliatus*								
Single flower number	0.483	< 0.001	0.085	< 0.001	0.549	< 0.001	0.340	< 0.001
Inflorescence number	0.323	< 0.001	0.312	< 0.001	0.516	< 0.001	0.146	< 0.001
Seed set	0.221	< 0.001	0.342	< 0.001	0.583	< 0.001	0.049	< 0.001

In *M*. *spicatum*, both absolute sexual and asexual biomass increased with increase in eutrophic levels. The ratio of asexual reproduction also increased with increase in eutrophic conditions, although sexual reproduction decreased (Fig 2b, Table 3). *M*. *spicatum* flower number per inflorescence increased with increase in eutrophic levels, although there was a decrease in number of inflorescence. Seed set increased with increase in eutrophic levels (Fig 3b, Table 4). The relationship between reproductive components and environmental variables indicated that the asexual biomass, sexual biomass, ratios of sexual/asexual reproduction, flower and seed production were all correlated with the four environmental variables (TN, TP, Chla and Secchi depth). Asexual biomass and ratio of asexual reproduction increased while sexual biomass and ratio of sexual reproduction decreased with increase in TN, TP and Chla. Sexual biomass and ratio of sexual reproduction increased with increase in Secchi depth, while the asexual biomass and ratio of asexual reproduction decreased (Fig 4b, Table 5). Flower number per inflorescence decreased, while inflorescence number and seed set increased with increase in the four environmental variables (TN, TP, Chla and Secchi) (Fig 5b, Table 6).

In *P*. *perfoliatus*, both absolute sexual and asexual biomass, as well as ratios of reproduction hardly varied across different eutrophic levels (Fig 2c, Table 3). Flower and seed production in *P*. *perfoliatus* varied, with flower number per inflorescence increasing while inflorescence number decreased with increase in nutrient levels. Seed set also decreased with increase in nutrient level (Fig 3c, Table 4). A comparison of reproductive components and environmental variables revealed that the absolute sexual and asexual biomass, together with the ratios of sexual and asexual reproduction, were minimally altered by the four environmental variables (TN, TP, Chla and Secchi depth) (Fig 4c, Table 5). However, the reproductive components changed with these four environmental variables. The single flower number increased while the inflorescence number and seed set decreased with the four environmental variables (Fig 5c, Table 6).

## Discussion

Variable reproductive allocation facilitates plant adaptability to different environments [29]. Based on variable eutrophic levels (TLI index) and the four environmental variables (TN, TP, Chla, Secchi), in the present study, *S*. *pectinata* and *M*. *spicatum* were common in eutrophic waters and exhibited greater plasticity in reproductive allocation, compared to *P*. *perfoliatus*, which was only found in clear waters.

Plant reproduction, including asexual and sexual reproduction, require greater resources, not only for their current growth but also to guarantee greater chances of seed set, or shoot fragments surviving and taking part in future population expansion [30–31]. Asexual reproduction is a vital feature for macrophytes in the aquatic habitat. It is suggested that asexual reproduction could increase on elimination of water stress, with all vegetative parts having the capacity to become propagules [2]. This claim could be supported by the present study.

In freshwater ecosystems, particularly in eutrophic environments, there is greater light attenuation and limited supply of inorganic carbon, which are critical factors limiting productivity of submerged macrophytes [32]. Asexual reproduction increased (including biomass and ratios allocated to asexual reproduction) with increase in eutrophic levels in *S*. *pectinata* and *M*. *spicatum*. The environmental variables, including TP, TN, and Chla, also had positive correlations with asexual reproduction because the plants require resources for their growth and more fragments for asexual reproduction. TP and TN are essential for plant growth. Although the relationship between three of the environmental variables and the plants’ asexual biomass correlated in two species, the effect Secchi depth, which is presumed to be biggest driver of change in light environment, varied between the two species.

*M*. *Spicatum* allocated more resources to asexual reproduction in lower Secchi depths. This could facilitate growth and access to the lake surface to obtain light and increase photosynthesis [33–34]. In addition, this could facilitate the individuals’ acquisition of CO_2_ directly from the atmosphere and overcome inorganic carbon limitation [33–34]. In this species, increase in shoot length also improves chances of asexual reproduction through the action of greater volumes of fragments. Population expansion may be triggered by disturbances from waves, invertebrates, fish, or anthropogenic activity. This is thought to be principal mode of dispersal for aquatic plant species [35]. Conversely, in *S*. *pectinata*, Secchi depth had marginal effects on plant asexual biomass, which indicates that the species experienced no light limitation for asexual acquisition. According to our observations, the plants set more branches instead of increasing the number of shoots which did not need to grow to the surface to obtain more light. This ensured that the plants had greater chances to set fragments from both branches and shoots in the species.

Reproductive allocation also varied among sexual components in *S*. *pectinata* and *M*. *spicatum*. Generally, the more limiting nutrients are in an environment, the more resources will be allocated to sexual reproduction, since sexual reproduction could guarantee genetic variation and recombination, which may increase survivorship in the plants in the future [19, 23, 36]. However, in our results, sexual ratio decreased with increase in eutrophic levels (including the TLI index, TN, TP, and Chla) in *S*. *pectinata* and *M*. *spicatum*, particularly in eutrophic lakes. This may due to two reasons. First, total resource acquisition of the plants could have remained constant, since, an increase in sexual reproduction leads to a decrease in asexual reproduction. This is very common in aquatic plants species [1, 37–38]. The other reason could be that the vegetative propagules confer more advantages than the seeds. In aquatic ecosystems, asexual reproduction is more critical compared to sexual reproduction [2]. The water environment, waves, and relatively stable temperatures facilitate vegetative reproduction. In addition, the prolific nature of vegetative propagules makes them more suitable for the spread and establishment of new populations [35]. Although seed can also spread by waves, there are higher resource requirements for each seed and greater risks associated with seedling establishment in high nutrient lakes.

The present study also reveals different effects of Secchi depth between *S*. *pectinata* and *M*. *Spicatum*. In *M*. *Spicatum*, sexual reproduction decreased with decrease in Secchi depth in the high nutrient lakes. This is because lower light acquisition leads to lower seed germination and seedling establishment. The plants prefer setting more vegetative propagules for the future [2]. In *S*. *pectinata*, however, Secchi depth had no effect on sexual reproduction. This indicates that light was not a limiting factor for seed set or flower production. This may be attributable to reproductive assurance in the future in the changing environment.

Unlike in *S*. *pectinata* and *M*. *Spicatum*, the available data for *P*. *perfoliatus* demonstrates stable reproductive allocation capacities, which is attributable to its lower plastic resource acquisition capacity, although the clear lakes are fewer in our study. We also find no differences in the TN, TP, Chla and Secchi depth among the various sampling sites. Photosynthesis in the species in clear stable environments occurs relatively uninhibited compared to in the other species under study. In addition, our previous studies have revealed that water depth as opposed to sediment nutrients is the limiting factor for the species [33]. In this study, all materials were collected at a depth of 1 m, which facilitated stable reproductive allocation patterns in the species. The other reason should be the tolerance to nutrient limited environments, or deficiency of a limiting nutrient such as N or P, since the study established relatively low N and P concentrations in its habitats. This is supported by our earlier study [33]. Thirdly, it could be attributed to the plant’s relatively low capacity to alter nutrient acquisition in its environment. According to our results of the field investigation, *P*. *perfoliatus* exhibited a lower capacity to alter its resource acquisition pattern. There were large populations of the species in the clear lakes. These individuals had adequate flower numbers, in addition to chlorophyll fluorescence (Fv/Fm) values that indicated uninhibited growth and photosynthetic activity in the clear water conditions (Liu et al., unpublished data).

While the greater emphasis placed on asexual reproduction over sexual reproduction is supported by the current study, what is the relationship between sexual reproduction and higher eutrophic environmental variables? Sexual reproduction could increase plant survivorship in future [36, 39]. Our results reveal that seed set and inflorescence number increase with increase in TN, TP and Chla, in *S*. *Pectinata*, *M*. *spicatum* and *P*. *Perfoliatus*. In the lower Secchi depths, the plants exhibit higher capacity for seed set. We, therefore, attribute this reproductive assurance strategy to higher eutrophic environment where nutrition levels are higher and the Secchi depths are lower. Generally, aquatic environments are relatively stable compared to terrestrial environments, so that asexual reproduction is the principal mode of propagation. Nevertheless, most of the environmental variables exhibit continuity and small-scale heterogeneity, establishing a dynamic that recurs on regional scales [40]. A short-term extreme event could illustrate the gross shortcomings of vegetative propagation strategies and their inability to withstand unsustainable conditions. In such cases, seeds are critical for population maintenance [2]. Flower inflorescences also represent shoot fragments. Dispersal of fragments such as inflorescences facilitates greater spread of seeds over long distances. Such modes of sexual reproduction could aid plants in overcoming the limits associated with extreme events in the future.

Among the three macrophytes species, *S*. *pectinata* and *M*. *spicatum* exhibited greater plasticity in allocation patterns in the eutrophic environments. They increased sexual and asexual reproduction, and their capacity to set offspring in future, effectively increasing their fitness in higher eutrophic conditions. *P*. *perfoliatus* also exhibited a capacity to adopt varied sexual reproductive strategies such as setting more offspring for the future, although only in clear conditions with low nutrient levels. Although adaptability in highly eutrophic lakes is more complex, including tolerance to high nutrient toxicity, and interactions with other organisms and physical conditions, our research provides direct evidence of variable reproductive allocation patterns, and how some species are able to survive in highly eutrophic lakes.
